# Efficacy of a multivalent vaccine against *Fasciola hepatica* infection in sheep

**DOI:** 10.1186/s13567-021-00895-0

**Published:** 2021-01-28

**Authors:** Rafael Zafra, Leandro Buffoni, Raúl Pérez-Caballero, Verónica Molina-Hernández, María T. Ruiz-Campillo, José Pérez, Álvaro Martínez-Moreno, Francisco J. Martínez Moreno

**Affiliations:** 1 Animal Health Department (Parasitology and Parasitic Diseases), Faculty of Veterinary Medicine, University of Córdoba, Sanidad Animal Building, Rabanales Campus, Córdoba, Spain; 2 Department of Anatomy, Comparative Pathology and Toxicology, Faculty of Veterinary Medicine, University of Córdoba, Sanidad Animal Building, Rabanales Campus, Córdoba, Spain

**Keywords:** *Fasciola hepatica*, Vaccine, Sheep, Protection

## Abstract

In this work we report the protection found in a vaccination trial performed in sheep with two different vaccines composed each one by a cocktail of antigens (rCL1, rPrx, rHDM and rLAP) formulated in two different adjuvants (Montanide ISA 61 VG (G1) and Alhydrogel^®^(G2)). The parameters of protection tested were fluke burden, faecal egg count and evaluation of hepatic lesions. In vaccinated group 1 we found a significant decrease in fluke burden in comparison to both unimmunised and infected control group (37.2%; *p* = 0.002) and to vaccinated group 2 (Alhydrogel^®^) (27.08%; *p* = 0.016). The lower fluke burden found in G1 was accompanied by a decrease in egg output of 28.71% in comparison with the infected control group. Additionally, gross hepatic lesions found in vaccine 1 group showed a significant decrease (*p* = 0.03) in comparison with unimmunised-infected group. The serological study showed the highest level for both IgG1 and IgG2 in animals from group 1. All these data support the hypothesis of protection found in vaccine 1 group.

## Introduction

Fasciolosis caused by the liver fluke *Fasciola hepatica* is widespread worldwide with presence in more than 81 countries [1]. It has a wide range of hosts, ungulates and other mammals including humans and it is considered as an emerging zoonotic disease by the World Health Organisation (WHO) [2]. Fasciolosis supposes a major problem in farming industry since it is of particularly importance in livestock, specially ruminants in which the disease is responsible for substantial economic losses estimated at 3.2 US$ billion/year [3]. These costs are due to both losses in production (i.e. milk, carcass composition as well as a delay to reach an appropriate slaughter weight) and treatment with anthelmintic drugs [4–6].

Traditionally, the control of the disease has been carried out using flukicides together with an adequate pasture management. Nevertheless, the incorrect use of such drugs resulted in the development of the anthelmintic resistance phenomena [7–12]. Simultaneously, there is a growing concern in consumers about the presence of chemical residues in animal products and the presence of these residues often force farmers to have withdrawal periods before animal products could be consumed.

Taking all issues into account the search of an effective vaccine seemed to be the best option [13]. Thus, during the last three decades, several researchers have been trying to identify molecules from the parasite with antigenic capacity as vaccine candidates [4, 13, 14]. Some of these vaccination trials have been conducted using only one antigen, a mixture of two antigens or, in recent years, a cocktail of molecules composed by more than two antigens mixed with adjuvants to help boost the immune system [15–19]. Nevertheless, results have shown variable results in terms of protection [15, 20–24] and consequently, to date, there is no vaccine formulation sufficiently efficient to reach commercial development. This is probably because *Fasciola hepatica* immunomodulates the host’s immune response toward a non-protective profile [25], even from early stages of the infection [14, 25–27].

In this work we report the results of a vaccination trial performed in sheep using two different vaccine candidates, each one composed by a cocktail of *F. hepatica* recombinant molecules formulated in different adjuvants.

## Materials and methods

### Experimental design and vaccine preparation

Thirty-seven 8-month-old male Merino-breed sheep obtained from a liver-fluke free farm were used for this study. Prior to commencing the trial, all animals were tested for parasite eggs three times at 4 days intervals by zinc-sulphate-based flotation technique, and were also serologically tested for *F. hepatica* specific antibodies by ELISA, with negative results in all cases. During the experiment, animals were housed indoors in the experimental farm of the University of Córdoba and fed with hay and commercial pellet.

Sheep were randomly distributed into four groups. Groups 1 and 2 (*n* = 10 each) were immunised subcutaneously, twice, 4 weeks apart, with two different vaccine formulations which included *F. hepatica* recombinant cathepsin L1 (rCL1), *F. hepatica* recombinant peroxiredoxin (rPrx), *F. hepatica* recombinant helminth defence molecules (rHDM) and *F. hepatica* recombinant leucine aminopeptidase (rLAP), plus the adjuvants Montanide ISA 61 VG (G1) (Seppic, Puteaux, France) and Alhydrogel^®^ 2% (G2) (InvivoGen, San Diego, CA, USA), respectively. For each vaccine dose, 100 µg of each antigen were used and mixed with 1 mL of adjuvant. Group 3 (*n* = 10) was not immunised and was orally challenged and group 4 (*n* = 7) was neither immunised nor infected and remained as negative control group.

For vaccine preparation, rCL1 (FHU62288) was generated by expression of the cDNA in *Saccharomyces cerevisiae* as previously described [28], expression and purification of rHDM (F6KNY7) was conducted in *E. coli* [29], rLAP (Q17TZ3) was obtained by cloning the cDNA in frame in BamHI and BglII sites of linearized pThio HisC *E. coli*, as previously described [30], and rPrx (U88577) was obtained by inserting the cDNA into the pPRO Ex HtA vector (Life Science Market-Gentaur Ltd. Hertfordshire, UK) and used to transform *E. coli* BL21-DE3 [31].

Four weeks after the second immunisation animals from group 1, 2 and 3 were experimentally infected with 150 metacercariae of the South Gloucester strain of *Fasciola hepatica* (Ridgeway Research Ltd, UK). On week 15 post-infection, euthanasia was conducted by intravenous injection of T61^®^ (MSD Animal Health, Salamanca, Spain) according to manufacturer’s instruction.

### Fluke burden and egg output

For fluke burden evaluation, the liver was removed from each animal during necropsy, the gallbladder was opened using a blunt scissor and carefully examined for the presence of flukes. Then, bile ducts were cut and opened, and flukes were recovered. Finally, the liver was cut into small pieces of 1 cm-side and placed into warm water (40 °C) for 30 min to collect the remaining flukes. All flukes were counted and measured (length and width) and the results were expressed as mean ± SD. From 8^th^ week after infection (wai) to the end of the experiment (15^th^ wai), faecal samples were collected individually from each sheep, weekly, and faecal egg counts (FEC) were performed by zinc-sulphate-based flotation technique. Briefly, three grams of faeces were mixed with zinc-sulphate solution. Then, the solution was placed in a McMaster chamber and eggs were counted, with a sensitivity of 25 eggs per gram (EPG). Results were expressed as cumulative EPG.

### Gross pathology

At necropsy, the liver was removed and photographed on the visceral and diaphragmatic surface for gross evaluation. The gross lesions observed in liver were scored separately by two pathologists in a blind way according the score showed in Table 1. Additionally, tissue samples from the left and right hepatic lobe were collected and fixed in 10% buffered formalin, embedded in paraffin wax and 4 µm thick sections were stained with the haematoxylin–eosin method for histopathological evaluation (data not shown).

**Table 1:** Score system to evaluate hepatic gross lesions in sheep

Score	Gross pathology
0	Absolutely no pathology evident, liver normal colour, consistency, and no visible signs of fluke lesions
1	Small areas of scar tissue and lesions, < 5% of the liver affected
2	Moderate areas of scar tissue and lesions, occurring in 5–10% of the liver
3	Moderate areas of scar tissue, thickening of bile ducts, small to moderate areas of necrosis, pus, 10-20% of liver affected
4	Moderate to large areas of scar tissue, thickened bile ducts evident. Moderate areas of necrosis, pus, haemorrhage, 20-30% of liver affected
5	Large areas of scar tissue, thickened bile ducts. Multiple necrotic foci, pus, haemorrhage, severe degeneration and > 30% of the total liver affected

### Antibody detection

Humoral immune response was analysed by detection of specific antibodies against *F. hepatica* (rCL1, rHDM, rPrx and rLAP), using the ELISA method. Briefly, 96 well microtiter plates (Sarstedt, Nümbrecht, Germany) were coated with 1 μg/mL of the corresponding antigen diluted in 0.05 M carbonate–bicarbonate buffer pH 9.6 (100 μL/well), and incubated overnight at 37 °C. After five washes with PBS 0.05% Tween 20, 100 μL/well of blocking buffer containing 1% BSA diluted in PBS was added and incubated for 30 min at 37 °C. To detect IgG1, wells were washed and 100 μL/well of plasma diluted in blocking buffer was added and incubated at 37 °C for 30 min. Triple serial dilutions (starting at 1:100) were performed to determine endpoint titre. For IgG2 detection, 100 μL/well of plasma diluted at 1:10 was added to each well and incubated at 37 °C for 30 min. After washing, 100 μL/well of primary antibody diluted 1:5000 (mouse anti-bovine IgG1 and anti-bovine IgG2; 7500820–7500830 Cedi-Diagnostics), in blocking buffer was added and incubated at 37 °C for 30 min. After incubation, wells were washed and anti-mouse IgG-HRP was added at 37 °C for 30 min (AbD-Serotec, STAR13B). The plate was washed and 100 μL/well of tetramethylbenzidine (TMB-Sigma) were added and incubated at room temperature for 10 min. The reaction was stopped by adding 50 μL/well of 1 M sulfuric acid and optical density was measured at 450 nm using a microplate photometer (MultiskanTM FC, Thermo Scientific). Results were expressed as antibody titre − log10 − for IgG1, and as optical density for IgG2.

### Statistical analysis

Statistical analysis was carried out using JASP version 0.13.1 (JASP Team 2020) and GraphPad Prism version 8 (GraphPad Software Inc., San Diego CA, USA). Shapiro- Wilk test was applied to evaluate whether distributions were parametric. Comparisons between groups were made using the U Mann–Whitney test for non-parametric distributions and unpaired t test for parametric distributions. For serological study, the non-parametrical ANOVA Kruskall-Wallis test with Dunn’s post hoc test was carried out. Data from gross lesions were shown as ordinal values (ranged from 0 to 5) and were expressed as median with 25^th^ and 75^th^ percentiles (P25–P75). A *p* value < 0.05 was considered statistically significant.

## Results

### Fluke burden and faecal egg count

Data from fluke burden and faecal egg count are shown in Table 2. Fluke burden (expressed as mean ± SD) was 42.57 ± 12.34 for group 1; 58.38 ± 9.84 for group 2 and 67.78 ± 13.85 for group 3. Statistical study (unpaired t test) showed a significant decrease of the fluke burden in group 1 in comparison with the positive control (group 3) (37.2%; *p* = 0.002). In the same way, group 1 group showed a significant decrease in fluke burden in comparison with group 2 (27.08%; *p* = 0.016). A non-significant reduction of 13.8% of the fluke burden was observed in group 2 when compared to the control group 3.

**Table 2:** Results from fluke burden and faecal egg count (individually and cumulative per group)

	Fluke burden	FEC (individually)	FEC (cumulative)
Group 1	42.57 ± 12.34*^, †^	275 (150/425)	1925
Group 2	58.38 ± 9.84	425 (256.3/450)	3300
Group 3	67.78 ± 13.85	300 (125/450)	2700
Group 4	–	–	–

Individually faecal egg count is expressed as median with P25–P75. Fluke burden is expressed as mean ± SD.

*Statistical differences (*p* < 0.05) compared with group 3.

^†^Statistical differences (*p* < 0.05) compared group 2.

In respect to faecal egg count, overall, a high variability within each group was observed. The cumulative FEC at the end of the experiment showed a total of 1925, 3300, 2700 epg for group 1, group 2 and group 3, respectively. When data were compared, no statistical difference was observed between groups. The comparative analysis showed a decrease in the cumulative FEC of 28.71% in group 1 in comparison with the control group. No parasites nor liver fluke eggs were detected in the negative control group 4.

### Gross pathology

Gross hepatic lesions consisted of scars and tortuous white tracts, affecting mainly the hepatic left lobe and in a lesser degree the right and quadrate hepatic lobes as well as enlargement of gallbladder and bile ducts. High variability was found in all groups. Results from gross pathology are shown in Table 3. The statistical analysis showed a significant decrease in group 1 score (median 1: CI 95% P25(1)-P75(3) *p* = 0.03) in comparison with group 3 (median 3: CI 95% P25(2.5)-P75(4.5)). As commented above, there were no statistical differences between group 1 and group 2 (median 3: CI 95% P25(3)-P75(4)). No statistical differences were found between group 2 in comparison with group 3.

**Table 3:** Results from gross pathology

	Gross Pathology	Light lesions	Moderate-to-severe lesions	Very severe lesions
Group 1	1 (1/3)^*^	57.16%	42.85%	–
Group 2	3 (3/4)	14.30%	85.70%	–
Group 3	3 (2.5/4.5)	–	77.78%	22.22%
Group 4	–	–	–	–

Data are expressed as median with P25–P75.

^*^Statistical differences (*p* < 0.05) compared with group 3.

The score obtained were also expressed as percentage of Light (score 1), Moderate-to- severe (scores 2–4), and very severe lesions (score 5). These results are also showed in Table 3. According to the score, group 1 showed mostly light gross lesions (57.15%) and in a lesser degree moderate to severe lesions (42.85%). On the other hand, most of the lesions (85.70%) observed in group 2 were moderate to severe and the rest were light (14.30%). Finally, the positive control group showed a 77.78% of moderate to severe lesions and was the only group with very severe lesions (22.22%). No lesions were observed in any of the animals from the control group 4.

### Humoral immune response

**Figure 1:**
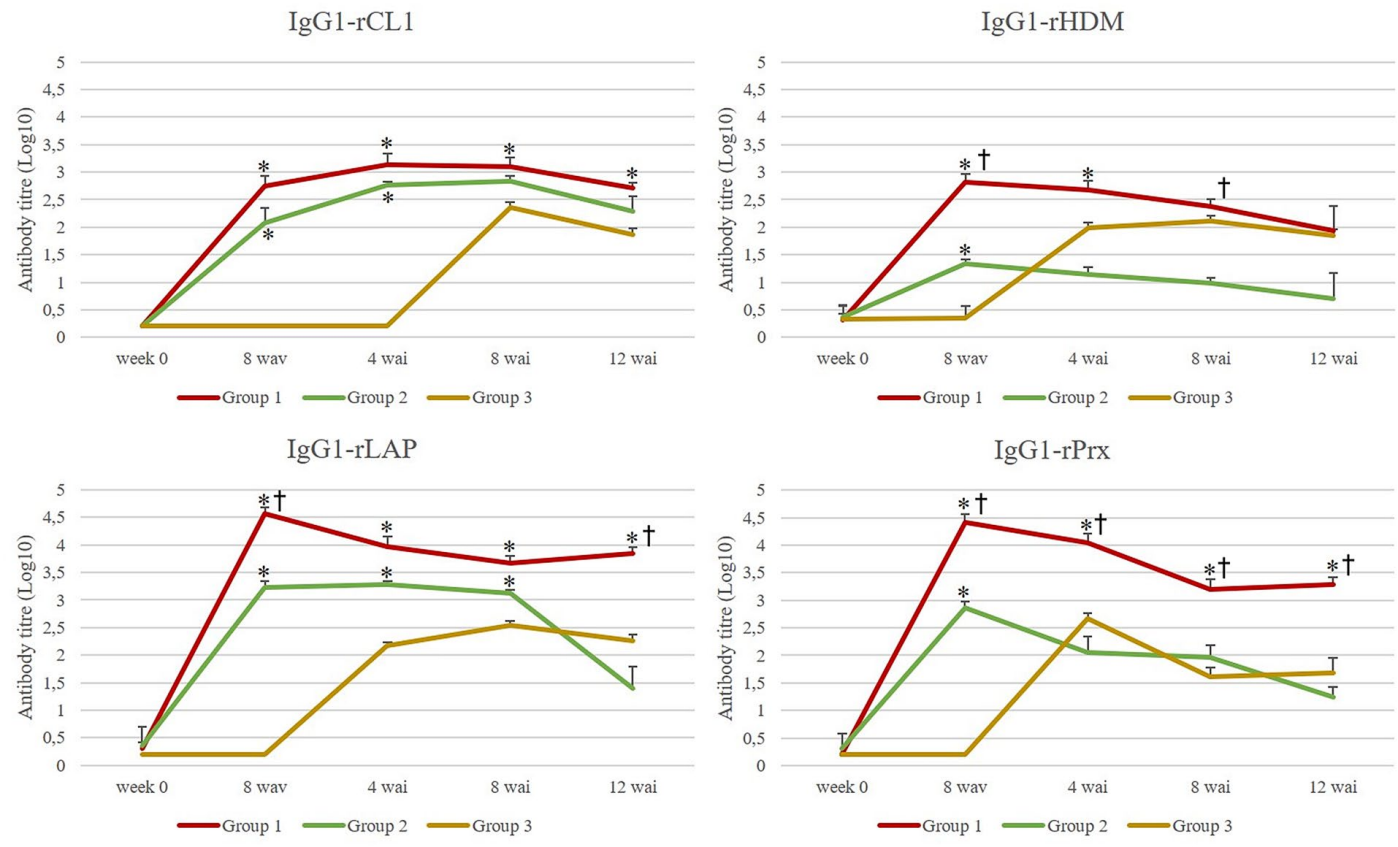
**Level of IgG1 against rCL1, rHDM, rLAP and rPrx for group 1 (antigens + Montanide), group 2 (antigens + Alhydrogel) and group 3 (positive control).** Each line represents the mean values obtained at each time point; bars indicate standard error. Asterisks indicate statistical differences (*p* < 0.05) in group 1 and 2, compared with group 3 and cross indicate statistical differences (*p* < 0.05) in group 1 compared with group 2.

**Figure 2:**
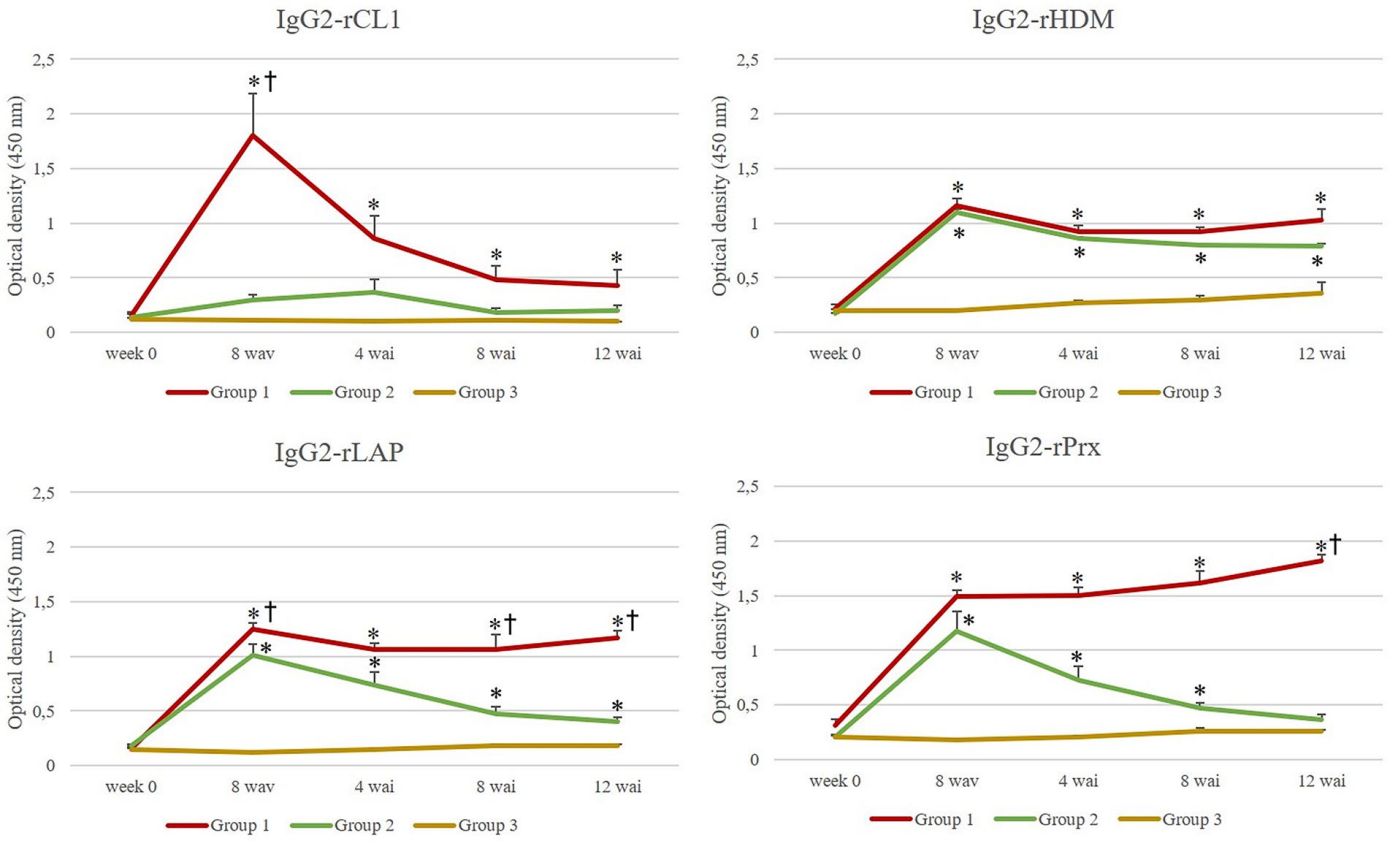
**Level of IgG2 against rCL1, rHDM, rLAP and rPrx for group 1 (antigens + Montanide), group 2 (antigens + Alhydrogel) and group 3 (positive control).** Each line represents the mean values obtained at each time point; bars indicate standard error. Asterisks indicate statistical differences (*p* < 0.05) in group 1 compared with group 3 and cross indicate statistical differences (*p* < 0.05) in group 1 compared with group 2.

Serological study was carried out at five timepoints during the experiment: week 0, 8 weeks after first vaccination (8 wav) and 4, 8 and 12 weeks after infection (wai). In this study the levels of IgG1 (Figure 1) and IgG2 (Figure 2) against rCL1, rHDM, rLAP and rPrx were analysed for all groups. No specific antibodies against any of the *F. hepatica* molecules were detected in the negative control group (group 4) at any of the time points analysed.

### Antibody response to rCL1

Both vaccinated groups showed a significant increase (*p* = 0.015 Group 1; *p* = 0.031 Group 2) of the IgG1 level after vaccination (8 wav) and a similar trend, though animals from group 1 displayed higher level during all time points. At 8 wav, there were no statistical differences for IgG1 between group 1 and 2. When data from group 1 were compared to the control group 3 for 4, 8 and 12 wai, significant differences were detected at all time points (Kruskall-Wallis *p* = 0.0001; Dunn’s *p* = 0.0002; Kruskall- Wallis *p* = 0.0027; Dunn’s *p* = 0.003; Kruskall-Wallis *p* = 0.0002; Dunn’s *p* = 0.001 respectively). Similarly, statistical differences were observed at 8 wav (*p* = 0.004) and 4 wai (*p* = 0.0006), when production of IgG1 from group 2 and 3 were compared. Production of IgG1 in control group showed a significant increase at 8 wai (*p* = 0.003), though level was lower compared to both vaccinated groups.

Production of IgG2 showed a significant increase in vaccinated group 1 at 8 wav (Mann–Whitney test *p* = 0.001) whereas a slight non-significant production was observed in group 2. In the positive control group, no production of IgG2 was observed during the trial.

### Antibody response to rHDM

Production of IgG1 showed a similar dynamic along the trial for both vaccinated groups. After vaccination, a significant increase was detected at 8 wav for group 1 and 2 (*p* < 0.0001 Group 1; *p* = 0.04 Group 2) in comparison to group 3. This was followed by a decreasing tendency in the level of antibodies until the end of the trial, though higher titres were observed in group 1 at all timepoints. In the positive control animals (group 3), production of IgG1 showed a sharp increase at 4 wai (*p* = 0.003) and a steady level throughout the course of infection. At 12 wai, there were not statistical differences between groups for IgG1.

Production of IgG2 increased significantly at 8 wav in vaccinated groups (*p* = 0.015 in both groups) and displayed a similar tendency after infection, though animals from group 1 showed a slight increase at the end of the trial. In positive control animals, no significant production was detected during the trial.

### Antibody response to rLAP

A significant production (*p* = 0.015) of IgG1 was observed right after vaccination in both immunised groups (8 wav), after which a decreasing dynamic was detected until the end of the trial. Similar to the observation for rCL1 and rHDM, IgG1 titres in animals vaccinated with *F. hepatica* molecules plus Montanide ISA 61 VG (group 1) exhibited the highest level during the experiment. In control group 3, significant IgG1 production was detected form 8 wai onwards (*p* < 0.0001 at 8 wai; *p* = 0.010 at 12 wai).

Likewise, IgG2 production was significantly elevated after vaccination in groups 1 and 2 (8 wav) (*p* = 0.015 in both groups) and showed the higher values in animals from group 1 at all timepoints. The comparative analysis exhibited significant differences between vaccinated groups at 8 wav (*p* = 0.019), 8 wai (*p* = 0.007) and 12 wai (*p* = 0.0006). No production of IgG2 was observed in control animals (group 3).

### Antibody response to rPrx

Overall, dynamic of both antibody isotypes showed similar results to those described above. Production of IgG1 was significantly increased after vaccination (8 wav) in group 1 (*p* = 0.015) and 2 (*p* = 0.015) being animals from group 1 those displaying the most elevated level throughout the course of the experiment. Control animals (group 3) produced specific-IgG1 from 4 wai onwards, though exhibiting a decreasing tendency.

A different dynamic was observed for production of IgG2 in both vaccinated groups. Although IgG2 was significantly elevated right after vaccination in all immunised animals (*p* = 0.015), sheep from group 1 exhibited an increasing trend in the IgG2 level in contrast to animals from group 2. No production was observed in the positive control animals.

## Discussion

During the last 30 years, the development of an effective vaccine against fasciolosis has been a challenging issue among researchers. As in the present study, combination of different molecules (cocktail vaccines, multivalent vaccines) has been previously analysed with variable results [14, 21, 32, 33]. However, the interaction between antigens in multivalent vaccines in fasciolosis is still unclear. Both synergy phenomena between antigens or competition have been suggested [34]. The use of a cocktail vaccine of recombinant proteins has demonstrated successful results in *Teladorsagia* infection in sheep, showing higher level of protection than individual antigens [35].

In this experiment, the vaccine cocktail was composed by four antigens with important functions in parasite’s life involved in migration, feeding, evasion of the immune response as well as with an immunomodulatory effect [21, 36]. Due to the role in crucial functions, cathepsins proteinases appeared as a promising candidate from the beginning [32, 37, 38]. Nevertheless, the results obtained with cathepsin L1 as vaccine antigen were variable depending on the host (cattle, goat, or sheep) as well as the type of cathepsin used (native or recombinant). The most promising results were obtained in cattle with native cathepsins L. It was previously reported in bovines vaccinated with native cathepsin L1 and cathepsin L2 + liver fluke haemoblobin of *F. hepatica* a significant decrease of the fluke burden (53.7% and 72.4% respectively), as well as a 98% anti-embryonation effect on eggs was found with CL2 + Hb vaccine [32]. Similarly, the same researchers observed by using the same *F. hepatica* molecules (CL2 + Hb), a significant increase in IgG1 levels as well as a positive correlation between these levels of immunoglobulins and the fluke burden when cattle, suggesting that it was possible to induce a protective response in cattle [38]. On the other hand, when recombinant antigens were applied, the use of adjuvant to boost immune response was required [22, 39, 40]. These studies performed with recombinant molecules and different adjuvants showed irregular results with a high variability in the protection rates found [14, 16, 39, 41, 42].

Leucine aminopeptidase (LAP) is involved in digestion, and possibly also invasion and migration through the hosts tissues [21, 33, 43]. Previous studies using recombinant LAP elicited parasite burden reduction of 49% and 89%, depending on the adjuvant used, though there was no reduction in the size of the parasites [21, 33]. LAP has been associated with other vaccine candidates to verify its effectiveness, both native and recombinant. In sheep, trials have been carried out with native cathepsins, CL1 and CL2 and LAP, both alone and in combination, with a reduction in the parasitic burden of 79% with the mixture of all three [33]. Moreover, the use of chimeric protein LAP + CL1 at different concentrations, induced protection with the maximum dose of the antigen (400 g) in relation to the parasitic burden (46.5%) [44]. These divergences between the level of protection reported and our results might be attributed to major differences in the vaccine formulation used, which may have led to the development of contrasting results in terms of vaccine efficacy. For instance, we recently reported significant B cell-based immune response differences in sheep when the same *F. hepatica* antigens were administered with different adjuvants, which resulted in diverse level of protection [24].

On the other hand, vaccination trials have recently been conducted using different fractions of HDM, CL1 and LAP in sheep, using different adjuvants. With synthetic HDM plus Quil A it was observed a delay in the production of antibodies and a slower growth of the liver flukes, as well as a lower production of antibodies [40]. Also, peroxiredoxin has been previously used in goats [15] and later in other host species in combination with LAP [45].

In our study, obtained results are indicative of certain level of protection and agree with previous studies carried out with native cathepsins, where similar indicators were tested [32, 44]. Group 1 (cocktail + Montanide ISA 61VG) showed a significant protection in terms of decrease of fluke burden (37.2%). There was no statistical difference between groups in cumulative FEC, probably due to the high variability within each group. Nevertheless, the comparative analysis revealed a decrease of 28.71% in cumulative FEC when group 1 was compared to the positive control. These results coincide with a recent report carried out in sheep with a chimeric protein composed by LAP and CL1 which showed protection expressed as reduction in fluke burden (25.5-46.5%) accompanied by a reduction in faecal egg count (22.7–24.4%) and higher titres of IgG1 and IgG2 [44]. These parasitological results are in accordance with the absence of very severe gross lesions as well as a decrease of 44.90% in moderate to severe gross lesions found in group 1 group in comparison with group 3. As a result, a significant decrease in gross lesions was found in group 1 in comparison with group 3. In addition, this decrease observed in gross pathology also corresponds to a significant decrease found in histopathological lesions compared again with group 3. In sheep from group 1, the presence of degenerated parasites and parasite eggs within the bile ducts as well as a different pattern in granulomatous lesions was observed. This finding supports the hypothesis of a certain degree of effective immune response against the parasite (data not shown). The differences in gross lesions between groups 1 and 2 did not reach the level of significance probably due to the high individual variability.

In our experiment, two adjuvants mixed with the same antigenic cocktail were assessed. It is well known that selection of an adequate adjuvant is crucial in terms of eliciting protection. In our trial we could not include an adjuvant-control group which might have helped to evaluate the adjuvant effect on the level of protection elicited in vaccinated animals. However, in some vaccine trials, a highly significant adjuvant effect could be demonstrated, as described using LAP as antigen, with a range of protection between 49.5% and 86.7% depending on the adjuvant used [21].

The significant protection observed in group 1 (Montanide ISA 61 VG), agrees with previous studies performed in mice and cattle where Montanide was used as adjuvant mixed with FhSAP2 in mice [46] and with rFhCL1 in cattle [39]. This protection could not be observed when aluminium adjuvant (Alhydrogel) was used. Although they have been used in many vaccines for over 90 years, there is still a controversy about the mode of action in ruminants, because most of the reports are based on in vitro studies [47]. In a recent study, the molecular signature activated by aluminium hydroxide in sheep has been described, resulting in the induction of endogenous danger signals, whose specific effects remains to be elucidated: it may contribute to long-lasting immune activation or to overstimulation of the immune system [48].

The serological study for IgG1 levels showed a significant increase for rCL1, rLAP and rPRx during all timepoints in group 1 in comparison with group 3. Group 2 showed a lower IgG1 levels compared with group 1 and significant increase in comparison with group 3. A similar pattern observed for IgG1 was also observed for IgG2. Thus, group 1 showed higher levels during the whole experiment, being significant in comparison with group 3. These results observed in group 1 agree with previous studies where high levels of antibodies were related with protection against *Fasciola hepatica* in ruminants [16, 21, 37, 44]. Furthermore, it was reported in sheep immunised with the same vaccine formulation of group 1, the recognition of different CL1 overlapping peptides from those observed in group 2 and 3, supporting the hypothesis of induction of immune protection with the combination of Montanide and cocktail antigen [24]. Similar results to those reported in sheep were also observed in cattle [49].

For rHDM, although higher titres of IgG1 were observed in group 1 compared with group 3, only significant differences were found at 8 wav and 4 wai, whereas this increase was significant for all timepoints for IgG2. It was observed, in vitro, supportive evidence that HDM play a key role for parasite survival [50–52] with potential as vaccine target.

In conclusion, the level of protection of a vaccine cocktail with two different adjuvants was assessed in sheep infected with *F. hepatica*. The combination with Montanide ISA 61 VG showed significant level of protection in comparison with unimmunised and infected group (37.2%) as well as a significant decrease in fluke burden and hepatic gross lesions. This group also showed a decrease of 28.71% in cumulative FEC. Finally, a strong humoral immune response was developed in animals with significant decrease of the fluke burden. These findings indicate certain level of protection conferred against *F. hepatica* with the combination of rCL1, rHDM, rPrx, rLAP and Montanide ISA 61 VG. Nevertheless, more studies are needed using multivalent vaccines against *F. hepatica* to understand the nature of interactions (synergies or competition) between antigens with the aim to find the appropriate vaccine formulation to confer an adequate immune protection.

## Data Availability

The datasets during and/or analyzed during the current study are available from the corresponding author on reasonable request.

## Abbreviations

rCL1: *Fasciola hepatica* recombinant cathepsin L1
rHDM: *Fasciola hepatica* recombinant helminth defence molecules
rLAP: *Fasciola hepatica* recombinant leucine aminopeptidase
rPrx: *Fasciola hepatica* recombinant peroxiredoxin
wai: Weeks after infection
wav: Weeks after first vaccination
FEC: Faecal egg count
EGP: Eggs per gram
TMB: Tetramethylbenzidine
SD: Standard deviation
